# New Precursors to 3-Sulfanylhexan-1-ol? Investigating the Keto–Enol Tautomerism of 3-*S*-Glutathionylhexanal

**DOI:** 10.3390/molecules26144261

**Published:** 2021-07-14

**Authors:** Jennifer R. Muhl, Lisa I. Pilkington, Rebecca C. Deed

**Affiliations:** 1 School of Chemical Sciences, The University of Auckland, Private Bag 92019, Auckland 1142, New Zealand; jmuh198@aucklanduni.ac.nz (J.R.M.); lisa.pilkington@auckland.ac.nz (L.I.P.); 2 School of Biological Sciences, The University of Auckland, Private Bag 92019, Auckland 1142, New Zealand

**Keywords:** 3-sulfanylhexan-1-ol, aroma compound precursors, white wine, deuterium exchange, tautomerism

## Abstract

The volatile thiol compound 3-sulfanylhexan-1-ol (3SH) is a key impact odorant of white wines such as Sauvignon Blanc. 3SH is produced during fermentation by metabolism of non-volatile precursors such as 3-*S*-gluthathionylhexanal (glut-3SH-al). The biogenesis of 3SH is not fully understood, and the role of glut-3SH-al in this pathway is yet to be elucidated. The aldehyde functional group of glut-3SH-al is known to make this compound more reactive than other precursors to 3SH, and we are reporting for the first time that glut-3SH-al can exist in both keto and enol forms in aqueous solutions. At wine typical pH (~3.5), glut-3SH-al exists predominantly as the enol form. The dominance of the enol form over the keto form has implications in terms of potential consumption/conversion of glut-3SH-al by previously unidentified pathways. Therefore, this work will aid in the further elucidation of the role of glut-3SH-al towards 3SH formation in wine, with significant implications for the study and analysis of analogous compounds.

## 1. Introduction

Volatile thiol compounds, such as 4-methylsulfanylpentan-2-one (4MSP), 3-sulfanylhexan-1-ol (3SH), and 3-sulfanylhexylacetate (3SHA) are significant contributors to the sensory profile of white wines, including those produced from *Vitis vinifera* L. cv. Sauvignon Blanc, imparting characteristic grapefruit and passionfruit aromas [1,2,3,4,5,6,7]. Despite the prominence of these odorants in the finished wines, these compounds are not found in appreciable levels in the grape berry or pressed juice and are produced almost exclusively during fermentation [8,9]. Currently only ~50% of the 3SH produced through fermentation can be explained through previously published pathways, allowing scope for more work to further enhance our understanding of the production of this impact odorant [9]. Additionally, studies on the role of 3SH in wine tend to focus on the biogenesis, synthesis, and applications with limited comments on the structure and reactivity of 3SH and its precursors.

Most studies on the production of 3SH focus on the longer-lived precursor species, such as 3*S*-glutathionylhexan-1-ol (glut-3SH) and 3*S*-cysteinylhexan-1-ol (cys-3SH), which were the first 3SH precursors to be identified [10]. Only recently, with the advance of analytical techniques, have more short-lived precursors such as 3*S*-cysteinylglycinylhexan-1-ol (CysGly-3SH) and 3*S*-γ-glutamylcysteinylhexan-1-ol (γ-GluCys-3SH) been identified and quantified in juice and wine samples [9,10].

One of the initial precursor compounds to 3SH produced in grapes and grape juice is 3-*S*-glutathionylhexanal (glut-3SH-al), formed by the Michael addition of glutathione to the α,β-unsaturated aldehyde, *E*-2-hexenal, upon the breakdown of fatty acids resulting from cell damage [8]. This detoxification process occurs both chemically and enzymatically and the aldehyde conjugate is subsequently transformed further into other precursors of 3SH and, through alcoholic fermentation by yeast, to 3SH [8,11,12].

Unlike other 3SH precursors, such as glut-3SH, the aldehyde functional group retained in glut-3SH-al gives the molecule increased reactivity, particularly in wine typical conditions. This reactivity has been observed previously under synthetic conditions, where attempts have been made to synthesise Cys-3SH-al. These attempts resulted in cyclised products, where the aldehyde functionality, as well as the alkene functionality, reacted with cysteine to produce mono and di-adducts [13]. Even once the conjugate with glutathione has been formed, the aldehyde functionality retains this reactivity. When glut-3SH-al was first identified in grape juice, it was identified in conjunction with the bisulfite adduct, glut-3SH-SO_3_, formed by the addition of a bisulfite ion to the aldehyde group [8,12].

The conversion of glut-3SH-al to 3SH is not yet well understood, with this precursor contributing to only 0.4% of total 3SH produced during fermentation in previous work [12]. It has been suggested that the bound form of glut-3SH-al as glut-3SH-SO_3_ impedes downstream metabolism to 3SH, resulting in the low conversion rates and forming an untapped sink of potential 3SH in the finished wine [8].

This group has been working to elucidate the kinetics of the reaction between glut-3SH-al and glut-3SH-SO_3_, and to further understand the resulting equilibrium. This investigation proceeded via NMR studies of these compounds and their interconversion. These analyses revealed artefacts in the proton and carbon NMR spectra, suggesting that glut-3SH-al is not present fully as the aldehyde form in aqueous solution.

This study aimed to explore the keto–enol tautomerization of glut-3SH-al to elucidate the characterisation and behaviour of glut-3SH-al in aqueous solutions. Previous work has indicated that the deuterium atoms in the H-2 position can be lost while in aqueous solution, resulting in a mix of deuteration of the internal standards, likely through enolization [9]. However, our elucidation of the behaviour of glut-3SH-al in ^1^H NMR suggests that the enol form is not a minor component of the solution, rather that the enol form is the predominant, and therefore more stable, form.

## 2. Results and Discussion

**Figure 1:**
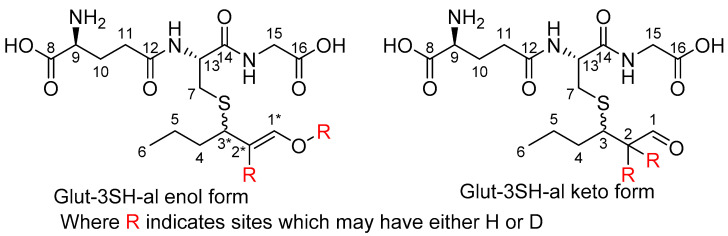
Glut-3SH-al as keto and enol tautomers. Where tautomers are distinguishable by NMR, the enol form is indicated by *.

The initial evidence of glut-3SH-al tautomerism was observed when conducting a time-course NMR investigation. This spectral change is likely due to both the equilibration of the tautomers in the aqueous solution and the exchange of enolizable protons for deuterium. The proton NMR spectra of glut-3SH-al is complex, as it is a mixture of two diastereomers (at C-3) as well as combinations of the recently identified tautomers (see Figure 1). As such, there are four species in solution, even before considering the effect of the gradual deuteration of C-2.

Compounds were synthesised using previously reported procedures [12,14] and characterisation was in agreement with values reported in the literature [12,14]. The recent identification of the additional structures present in solution required further interpretation of the characterisation data, but only the NMR data provided evidence of the additional structures.

**Figure 2:**
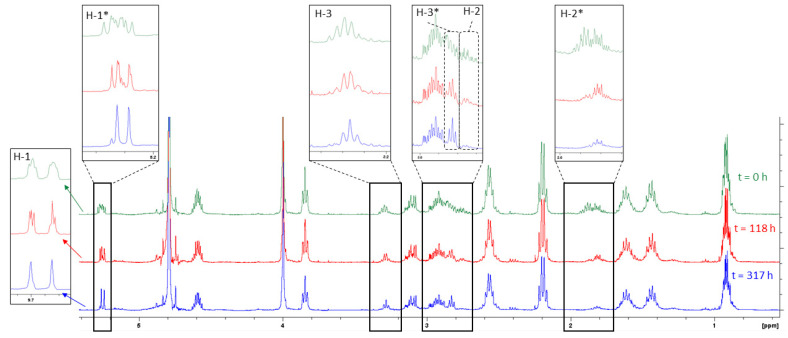
^1^H NMR spectra of glut-3SH-al in D_2_O over time. Where tautomers are distinguishable by NMR, the enol form is indicated by *.

To characterise the compounds by NMR, both 1D and 2D experiments were carried out on a solution of glut-3SH-al in D_2_O (approx. 80 mg mL^−1^). Additionally, ^1^H NMR spectra were obtained at various intervals to assess the change in the spectra over time (see Figure 2). The peak due to H-1*, the proton bonded to C-1 of the enol form of glut-3SH-al identified in this work, can be seen in the ^1^H NMR spectra of glut-3SH-al reported by Muhl et al. [14], but is outside the range of the ^1^H NMR spectrum shown in Thibon et al. [12].

Assignment of signals corresponding to H-6, H-5, H-4, and H-3 was confirmed by comparison of the NMR spectra of glut-3SH-al to the spectra of glut-3SH-al-*d*_8_, in which deuteriums occupy the H-6, H-5, H-4, and H-3 positions. This confirmed the assignment of the signals resulting from H-1 and H-2, as well as the signals resulting from protons on the glutathione moiety. Change in the multiplicity of the peaks due to H-1 and H-3, and a decrease in the integrals of peaks arising from H-2, over time was observed (see Figure 2) and taken as evidence of the deuterium exchange occurring as the enolization occurred.

The incorporation of two deuterium atoms at the H-2 position was confirmed by HRMS analysis of the sample of glut-3SH-al in D_2_O used for NMR analysis. After the sample in D_2_O was stored for two weeks, the HRMS spectrum showed a major peak at *m*/*z* 430.1587, corresponding to the sodium ion adduct of glut-3SH-al-*d*_2_ (C_16_H_25_D_2_N_3_NaO_7_S) while the peak at *m*/*z* 429.16 corresponding to the sodium ion adduct of glut-3SH-al-*d*_1_ was less than ¼ of the height of the peak due to glut-3SH-al-*d*_2_. Additionally, the largest of the peaks due to [M + H]^+^ was at *m*/*z* 408 for glut-3SH-al-*d_2_*, with the peak at *m*/*z* 407 due to glut-3SH-al-*d*_1_ being approximately 25% of the height of the peak at 408, and the peak at *m*/*z* 406 due to glut-3SH-al being negligible. Thus, after storing glut-3SH-al for 14 days in D_2_O the major species is glut-3SH-al-*d*_2_, meaning enolization has proceeded significantly.

**Scheme 1:**
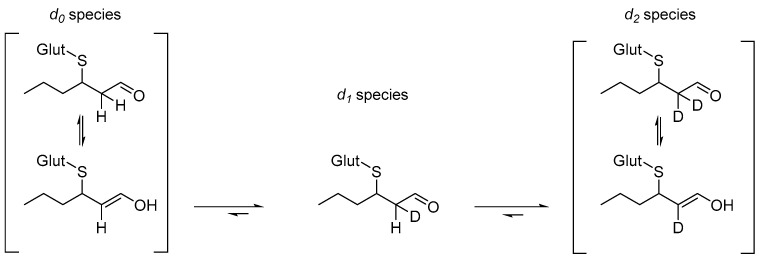
The key equilibria occurring in solutions of glut-3SH-al in deuterated solvents (adapted from Nichols and Waner 2010 [15]).

The incorporation of deuterium into the structure of glut-3SH-al over time was studied by monitoring the change of abundance of species in solution in a time-course analysis. Initially, the previously described NMR study of glut-3SH-al in D_2_O showed that over time, the protons in the H-2 position were eliminated, as shown in Scheme 1. This is to be expected, as the use of deuterated solvent means that the reaction from *d*_0_ to *d*_2_ species (see Scheme 1) is favoured and can effectively be considered as an irreversible reaction [15].

**Scheme 2:**
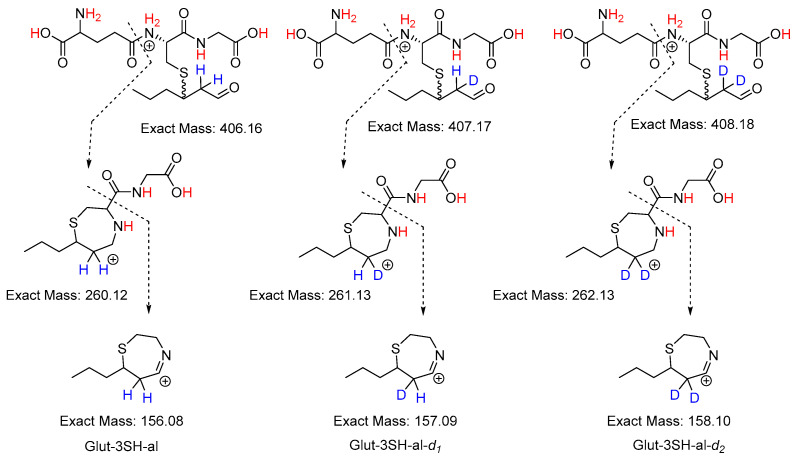
Proposed fragmentation of [M + H]^+^ to give the product ions monitored in QqQ analysis. Sites where rapid hydrogen deuterium exchange may occur are indicated in red. Sites where hydrogen–deuterium exchange may occur via the keto enol tautomerism monitored in this work are indicated in blue.

Further time-course studies were carried out by repeated analysis of a sample of glut-3SH-al in D_2_O by product ion mass spectrometry using a QqQ instrument. The ability of heteroatom-bonded protons (indicated in red in Scheme 2) to exchange for deuterium in solutions, or formation of [M + D]^+^ ions, results in many molecular ions possibly being present in solution. As such, the product ions studied were selected to avoid the impact of unwanted hydrogen–deuterium exchange on the results of the analysis. The product ions chosen for each monitored species, glut-3SH-al, glut-3SH-al-*d*_1_, and glut-3SH-al-*d*_2_, and the proposed fragmentation mechanisms are shown in Scheme 2.

Generally, the abundance of the *d*_0_ species decreased over time, while the abundance of the *d*_1_ and *d*_2_ species increased over time. Analysis of samples prepared in DCl in D_2_O showed a high concentration of the deuterated species at the first time point, suggesting that the tautomerism reached an equilibrium fairly rapidly at the lower pH.

This work demonstrates, for the first time, that glut-3SH-al can undergo keto–enol tautomerism resulting in hydrogen–deuterium exchange occurring at the C-2 position of the hexanal moiety. The NMR data also provide evidence that the enol form is not a minor component of the system, as is typical for mono-carbonyl compounds [16], rather that the enol form accounts for approximately 60% of glut-3SH-al, estimated by the relative integrals of the peaks due to H-1 and H-1*.

The findings presented here are critical factors in the study and understanding of volatile thiols and their precursors. The existence of the enol form of glut-3SH-al in wine-like solutions is likely to influence all other reactions that glut-3SH-al could undergo in solution. The reactivity of the enol form likely differs to the reactivity of the keto form and may even be the substrate for enzymatic reactions previously unidentified in wine. It is possible that glut-3SH-al is consumed through pathways other than reduction to glut-3SH, and even that these pathways result in previously unidentified precursors to 3SH, which may further elucidate 3SH biogenesis in wine.

To the best of our knowledge, this is the first report of the predominance of the enol form of glut-3SH-al in wine typical solutions. Future exploration of 3SH biogenesis should consider this tautomerism and how the enol form of glut-3SH-al may contribute to 3SH formation, possibly via previously unidentified pathways. There is also scope for exploring the keto–enol tautomerism of other carbonyl-containing volatile thiol precursors, and their influence on the aroma composition of the finished wines. 

## 3. Materials and Methods

### 3.1. General Methods

Commercial reagents were used without further purification unless otherwise stated. All ultrapure water came from a Barnstead Nanopure Diamond™ (ThermoFisher Scientific, Waltham, MA, USA) with resistivity at 18.2 MΩcm.

### 3.2. Analysis of Spectra

High-resolution mass spectrometry (HRMS) was carried out on a Bruker MicrOToF-QII instrument (Bruker, Bremen, Germany), coupled to an electrospray ionisation (ESI) source. NMR spectra were recorded as specified on a Bruker DRX400 spectrometer (Bruker, Bremen, Germany) (400 MHz for ^1^H nuclei and at 100 MHz for ^13^C nuclei). All chemical shifts (δ) are reported in parts per million (ppm) relative to D_2_O (4.79 ppm), as an internal reference.

QqQ mass spectrum data were obtained using an Agilent 6400 Triple Quadrupole mass spectrometer (Santa Clara, CA, USA) system equipped with an Agilent (Santa Clara, CA, USA) jet stream electron spray ionisation probe. Nitrogen (BOC, Auckland, New Zealand) was used as the desolvation gas at 9 L/min at 200 °C, the nebuliser was set at 45 psi, and the sheath gas temperature is 350 °C at 11 L/min. The ESI capillary was set at 4000 V and the nozzle voltage at 500 V. The optimised fragmentation voltage for glut-3SH-al and the deuterated analogues was 100 V.

### 3.3. Synthesis of 3-S-Glutathionylhexanal, Glut-3SH-al

Synthesis of glut-3SH-al was carried out following the procedures reported by Muhl et al. 2020 [10]. To a mixture of 2-*E*-hexenal (90 mg, 0.92 mmol) in water (10 mL, deionised) was added L-glutathione (220 mg, 0.72 mmol) and Cs_2_CO_3_ (125 mg, 0.39 mmol) before being placed under an atmosphere of nitrogen and stirred at room temperature for 20 h. The reaction mixture was washed with pentane:CH_2_Cl_2_ (9:1, 20 mL) and the aqueous layer was acidified with HCl (2M, aq) to pH 1–2. The resulting orange solution was flash frozen (N_2_, liquid) before freeze drying for 22 h. The result was an orange powder which was characterised to contain glut-3SH-al (0.413 g, 142%). Portions of crude product (40 mg) were purified on prepacked Supelclean™ ENVI™-18 SPE tubes, eluted with water (3 mL), 5% aqueous ethanol (3 mL), 15% aqueous ethanol (10 mL), and ethanol (3 mL). Elution of the product occurred during 15% ethanol and fractions containing the product were freeze-dried and the resulting white powder was characterised to be glut-3SH-al (9 mg, 23%).

### 3.4. Purification of Synthesised Compounds

Reverse-phase chromatography was carried out on prepacked Supelclean™ ENVI™-18 SPE tubes. Thin-layer chromatography (TLC) was carried out using Merck silica gel F254 aluminium plates pre-coated with silica. Solvents were used as specified. Compounds were visualised using ultraviolet fluorescence and/or staining with vanillin in ethanolic sulfuric acid (with heating).

δ_H_ (400 MHz; D_2_O) 0.89–0.94 (6H, m, H-6), 1.41–1.47 (4H, m, H-5), 1.58–1.63 (4H, m, H-4), 1.77–1.83 (1H, m, H-2*), 2.19 (4H, q, *J* = 7.2 Hz, H-10), 2.54–2.59 (4H, m, H-11), 2.75–2.95 (7H, m, H-2, H-3*, H-7), 3.08–3.13 (2H, m, H-7*), 3.29 (1H, m, H-3), 3.83 (2H, t, *J* = 6 Hz, H-9), 3.98 (4H, s, H-15), 4.56–4.62 (2H, m, H-13, H-13′), 5.24–5.30 (1H, m, H-1*), 9.67, 9.70 (1H, d, *J* = 12 Hz, H-1).

δc(100 MHz; D_2_O) 13.0, 13.1 (C-6, C-6*), 19.2, 19.4 (C-5, C-5*), 26.0 (C-10), 29.5 (C-2*) 31.1, 31.2, 31.3 (C-11, C-7′, C-7), 36.6, 36.7 (C-4, C-4*), 39.3 (C-3), 41.5, 41.6, 41.8 (C-2, C-3* C-15), 53.3, 53.4 (C-13, C-13′), 53.8 (C-9), 89.2 (C-1*), 172.4, 172.5, 173.7, 173.8, 174.8 (C-8, C-12, C-14, C-16), 206.3, 206.4 (C-1).

^1^H and ^13^C NMR were in agreement with that reported in the literature [8,10]. Where tautomers are distinguishable by NMR, the enol form is indicated by *. Where signals due to diastereomers were distinguishable, they are indicated by ’. Spectra can be found online in the Supplementary Materials.

## Data Availability

The data presented in this study are available in the text and in the Supplementary Materials.

## Supplementary Materials

The following are available online. ^1^H and ^13^C NMR spectra for synthesis of glut-3SH-al and HRMS data from NMR deuterium exchange experiment.

## Sample Availability

Samples of the compound 3*S*-glutathionylhexanal are available from the authors.
