# Supplementary material for: New Precursors to 3-Sulfanylhexan-1-ol? Investigating the Keto–Enol Tautomerism of 3-S-Glutathionylhexanal
Source: Molecules. 2021 Jul 14;26(14):4261. doi: 10.3390/molecules26144261 (PMC8303116; doi:10.3390/molecules26144261)
Supplement: Supplementary file 1 [file molecules-26-04261-s001.zip › molecules-1278762-supplementary.pdf]

New Precursors to 3-Sulfanylhexan-1-ol? Investigating the Keto-Enol Tautomerism of 3-*S*-Glutathionylhexanal

Jennifer R. Muhl [a]
Lisa I. Pilkington [a]
Rebecca C. Deed *[a,b]
[a] School of Chemical Sciences, University of Auckland, Private Bag 92019, Auckland 1142, New Zealand.
[b] School of Biological Sciences, University of Auckland, Private Bag 92019, Auckland 1142, New Zealand.
* indicates the corresponding author.
rebecca.deed@auckland.ac.nz

Supporting Information

Contents

1. HRMS Spectra of Glut-3SH-al-d_2
2. ^{1}H NMR of Glut-3SH-al
3. ^{13}C NMR of Glut-3SH-al

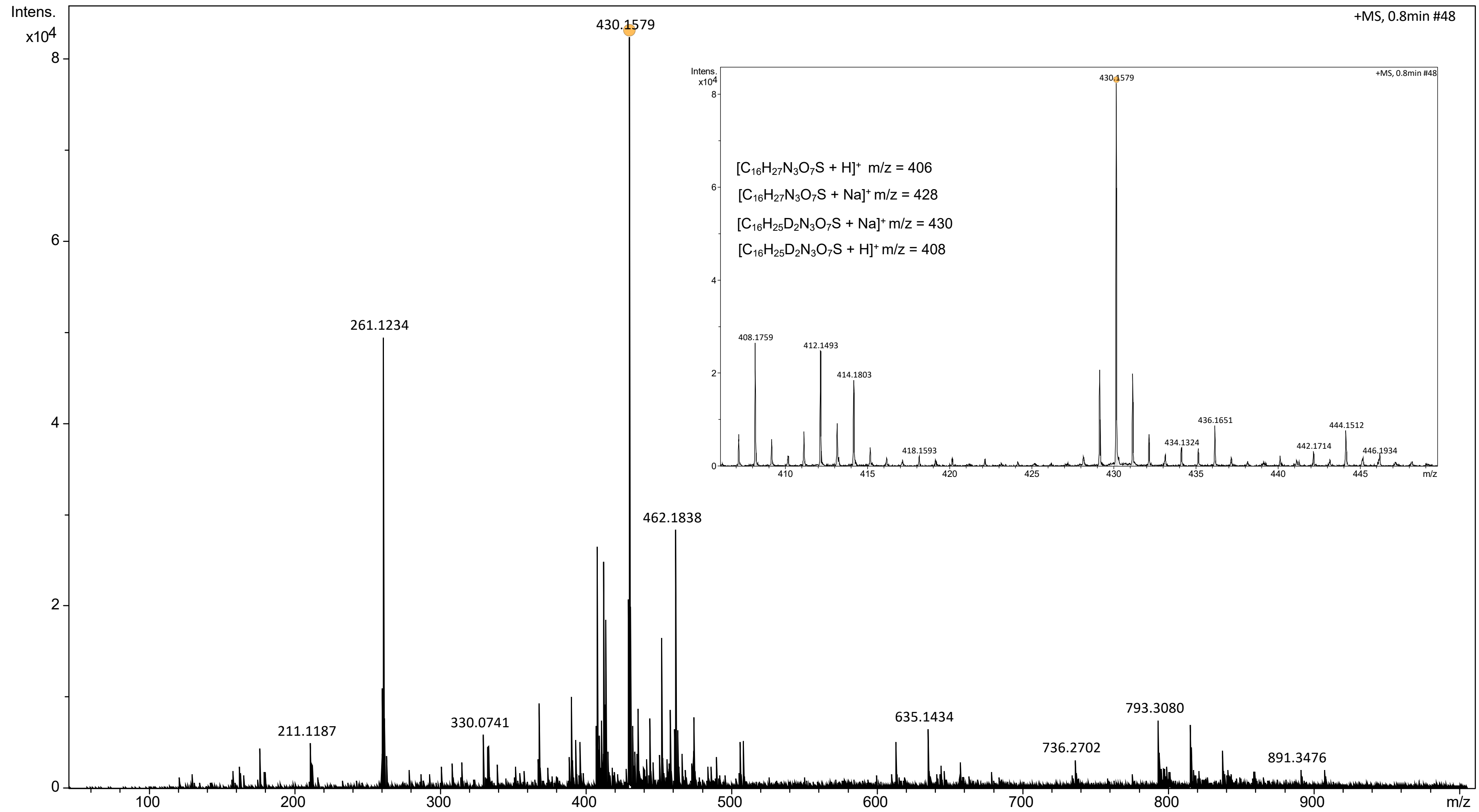

Acquisition Parameter

Source Type	ESI	Ion Polarity	Positive	Set Nebulizer	0.4 Bar
Focus	Active	Set Capillary	4500 V	Set Dry Heater	180 °C
Scan Begin	50 m/z	Set End Plate Offset	-500 V	Set Dry Gas	4.0 l/min
Scan End	1000 m/z	Set Collision Cell RF	150.0 Vpp	Set Divert Valve	Waste

Meas. m/z	#	Ion Formula	m/z	err [ppm]	mSigma	# Sigma	Score	rdb	e^- Conf	N-Rule
430.1579	1	$C_{16}H_{25}D_2N_3NaO_7S$	430.1587	-2.1	23.7	1	100.00	4.5	even	ok

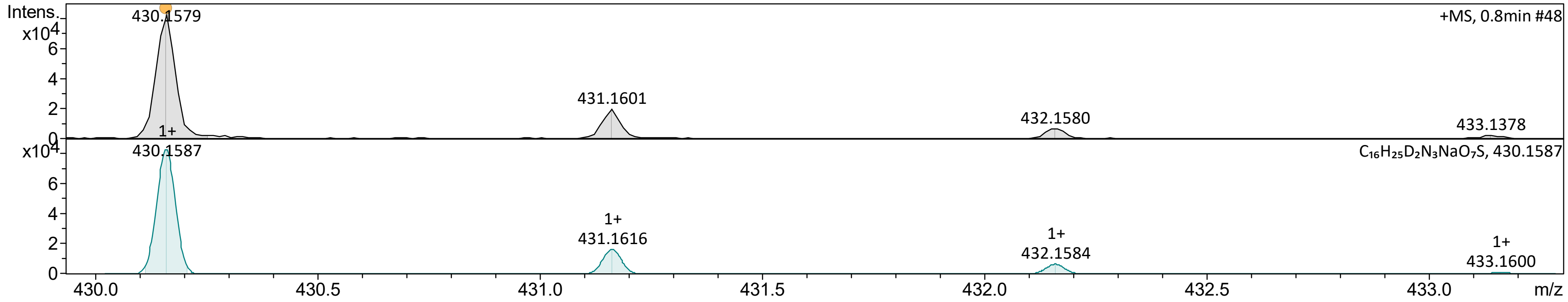

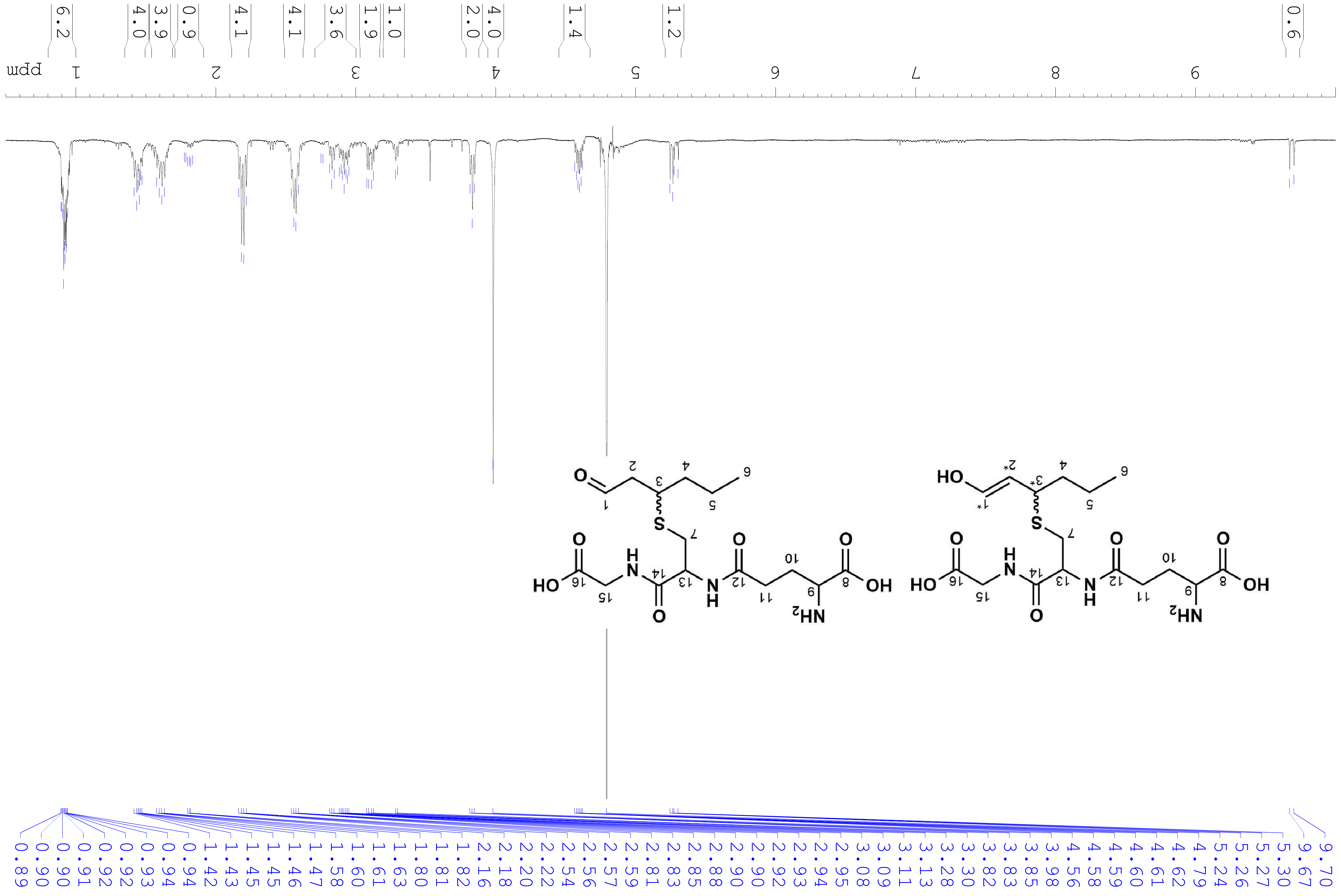
ppm
1
2
3
4
5
6
7
8
9
6.2
4.0
3.9
0.9
4.1
4.1
3.6
1.9
1.0
2.0
4.0
1.4
1.2
0.6
0.89 0.90 0.90 0.91 0.92 0.92 0.93 0.94 0.94 1.42 1.43 1.45 1.45 1.46 1.47 1.58 1.60 1.61 1.63 1.80 1.81 1.82 2.16 2.18 2.20 2.22 2.54 2.56 2.57 2.59 2.81 2.83 2.85 2.88 2.90 2.90 2.92 2.93 2.94 2.95 3.08 3.09 3.11 3.13 3.28 3.30 3.82 3.83 3.85 3.98 4.56 4.58 4.59 4.60 4.61 4.62 4.79 5.24 5.26 5.27 5.30 9.67 9.70

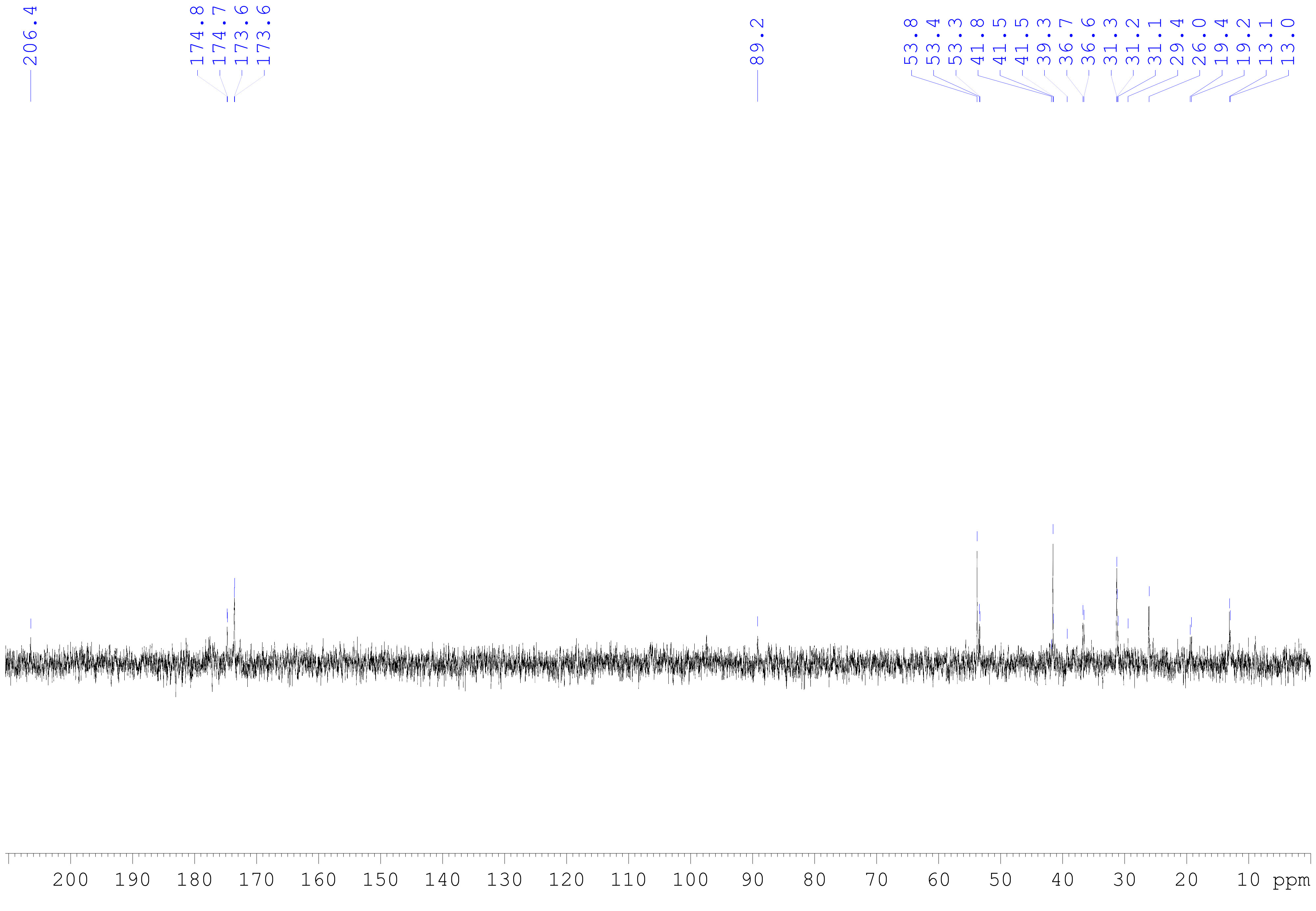
206.4
174.8
174.7
173.6
173.6
89.2
53.8
53.4
53.3
41.8
41.5
41.5
39.3
36.7
36.6
31.3
31.2
31.1
29.4
26.0
19.4
19.2
13.1
13.0
200
190
180
170
160
150
140
130
120
110
100
90
80
70
60
50
40
30
20
10
ppm
